# Nanoemulsions of synthetic rhamnolipids act as plant resistance inducers without damaging plant tissues or affecting soil microbiota

**DOI:** 10.3389/fpls.2023.1195718

**Published:** 2023-08-22

**Authors:** Milagro Mottola, María C. Bertolino, Lucille Tihomirova Kourdova, Jessica Aye Valdivia Pérez, María Florencia Bogino, Natalia E. Nocelli, Ludovic Chaveriat, Patrick Martin, Raquel V. Vico, Georgina Fabro, María Laura Fanani

**Affiliations:** ^1^ Centro de Investigaciones y Transferencia Tierra del Fuego (CIT-TDF) Consejo Nacional de Investigaciones Científicas y Técnicas (CONICET), Universidad Nacional de Tierra del Fuego (UNTDF), Rio Grande, Argentina; ^2^ Departamento de Química Orgánica, Facultad de Ciencias Químicas, Universidad Nacional de Córdoba, Córdoba, Argentina; ^3^ Instituto de Investigaciones en Físico-Química de Córdoba (INFIQC), Consejo Nacional de Investigaciones Científicas y Técnicas (CONICET), Córdoba, Argentina; ^4^ Departamento de Química Biológica Ranwel Caputto, Facultad de Ciencias Químicas, Universidad Nacional de Córdoba, Córdoba, Argentina; ^5^ Centro de Investigaciones en Química Biológica de Córdoba (CIQUIBIC) Consejo Nacional de Investigaciones Científicas y Técnicas (CONICET), Córdoba, Argentina; ^6^ Univ. Artois, UnilaSalle, Unité Transformations & Agroressources, Béthune, France

**Keywords:** nanoemulsions, surfactant, rhamnolipids, bioprotectant, synthetic, resistance-inducer, plant health, sustainable

## Abstract

Plant pathogens and pests can cause significant losses in crop yields, affecting food security and the global economy. Many traditional chemical pesticides are used to combat these organisms. This can lead to the development of pesticide-resistant strains of pathogens/insects and negatively impact the environment. The development of new bioprotectants, which are less harmful to the environment and less likely to lead to pesticide-resistance, appears as a sustainable strategy to increase plant immunity. Natural Rhamnolipids (RL-Nat) are a class of biosurfactants with bioprotectant properties that are produced by an opportunistic human pathogen bacterium. RL-Nat can act as plant resistance inducers against a wide variety of pathogens. Recently, a series of bioinspired synthetic mono-RLs produced by green chemistry were also reported as phytoprotectants. Here, we explored their capacity to generate novel colloidal systems that might be used to encapsulate bioactive hydrophobic compounds to enhance their performance as plant bioprotectants. The synthetic mono-RLs showed good surfactant properties and emulsification power providing stable nanoemulsions capable of acting as bio-carriers with good wettability. Synthetic RLs-stabilized nanoemulsions were more effective than RLs suspensions at inducing plant immunity, without causing deleterious effects. These nanoemulsions were innocuous to native substrate microbiota and beneficial soil-borne microbes, making them promising safe bio-carriers for crop protection.

## Introduction

Bioprotectants are a sub-type of biostimulants, i.e.: complex mixes of biological products -such as essential plant nutrients, plant growth regulators, or plant protective compounds- that improve plant productivity as a consequence of the novel or emergent properties of the complex of constituents and not as a sole consequence of their presence (Yakhin et al., 2017). It is well known that to counteract the deleterious effects of phytopathogens, plants have evolved a series of constitutive and inducible defense mechanisms (Spoel and Dong, 2012). Constitutive defenses are species-specific, multigenic and quantitative and thus difficult to manipulate genetically or biochemically. In contrast, inducible defenses, like those triggered by pathogen molecular patterns, defense hormones or elicitins, can generate local and/or systemic responses that do confer effective protection to many plants, including crops (Vallad and Goodman, 2004; Reglinski et al., 2014) against a wide variety of pathogens (virus, bacteria, fungi, oomycetes) with different lifestyles (biotrophs, hemibiotrophs, necrotrophs) (Li et al., 2011; Choi et al., 2012; Dafermos et al., 2012; Sanchez et al., 2012; Ouyang et al., 2015; Derevnina et al., 2016; Monnier et al., 2018; Platel et al., 2021).

The induction of plant innate immune responses involves the onset of various defense mechanisms, including a rapid influx of calcium and generation of reactive oxygen species (ROS) at the plasma membrane, followed by an extracellular alkalinization and the intracellular activation of mitogen-activated protein kinase cascades. ROS and phosphorylation cascades signal subsequent cellular events, such as the deposition of callose polymer reinforcing the cell wall, wide transcriptional changes of defense genes, biosynthesis of pathogenesis-related (PR) proteins, production of secondary metabolites such as phytoalexins or antimicrobial peptides, and others (Spoel and Dong, 2012). The activation of these defense responses can efficiently control most of the non-adapted plant pathogens.

The Rhamnolipids (RLs) are among the wide variety of natural compounds that can generate Induced Resistance (IR) in plants (De Kesel et al., 2021). They comprise a class of biosurfactants that contain rhamnose as the sugar moiety linked to β-hydroxylated fatty acid chains. There are two main classes of rhamnolipids: mono-rhamnolipids and di-rhamnolipids, which have one or two rhamnose groups, respectively. These glycolipids have been demonstrated to have a wide range of antibacterial and antifungal properties either directly or *via* the stimulation of the plant´s innate immune system (Abalos et al., 2001; De Jonghe et al., 2005; Perneel et al., 2008; Varnier et al., 2009; Sanchez et al., 2012; Monnier et al., 2018; Platel et al., 2022). The existence of a plant receptor for RLs has not been described yet; however, it has been reported that these molecules cause broad-spectrum basal resistance through a LORE-receptor independent pathway that is affected by the composition of plant plasma membrane sphingolipids, resulting in the activation of efficient immune responses (Schellenberger et al., 2021). Furthermore, the effect of natural RLs on plant cells has been related to a direct interaction with plasma membrane lipids, where their amphiphilic character and surface properties play a major role (Monnier et al., 2019).

Surfactants find applications in almost all chemical industries, but their use may cause pollution and toxic effects on living organisms. These concerns have led to the development of innovative and environmentally friendly surfactants. RLs show a high potential to be used in areas such as agriculture since they are considered as easily biodegradable and exhibit low ecotoxicity (Randhawa and Rahman, 2014). Additionally, natural Rhamnolipids (RL-Nat) are excellent emulsion stabilizers, which turn out this surfactant family subject of widespread interest in the biotechnology (Lovaglio et al., 2011). RL-Nat are mostly produced using *Pseudomonas aeruginosa*, a bacterial species considered an opportunistic human pathogen. Therefore, it cannot be used as a biocontrol agent. Furthermore, large-scale production of RL-Nat is difficult because low yields are obtained from this bacterium. To overcome these issues, the design and synthesis of a series of mono-RLs intending a Green Chemistry approach was recently developed. As an additional advantage, the chemical synthesis of RLs is easy to scale-up at industrial level with affordable costs when compared to the complicated and high cost RL-Nat purification (Robineau et al., 2020; Platel et al., 2021).

**Figure 1:**
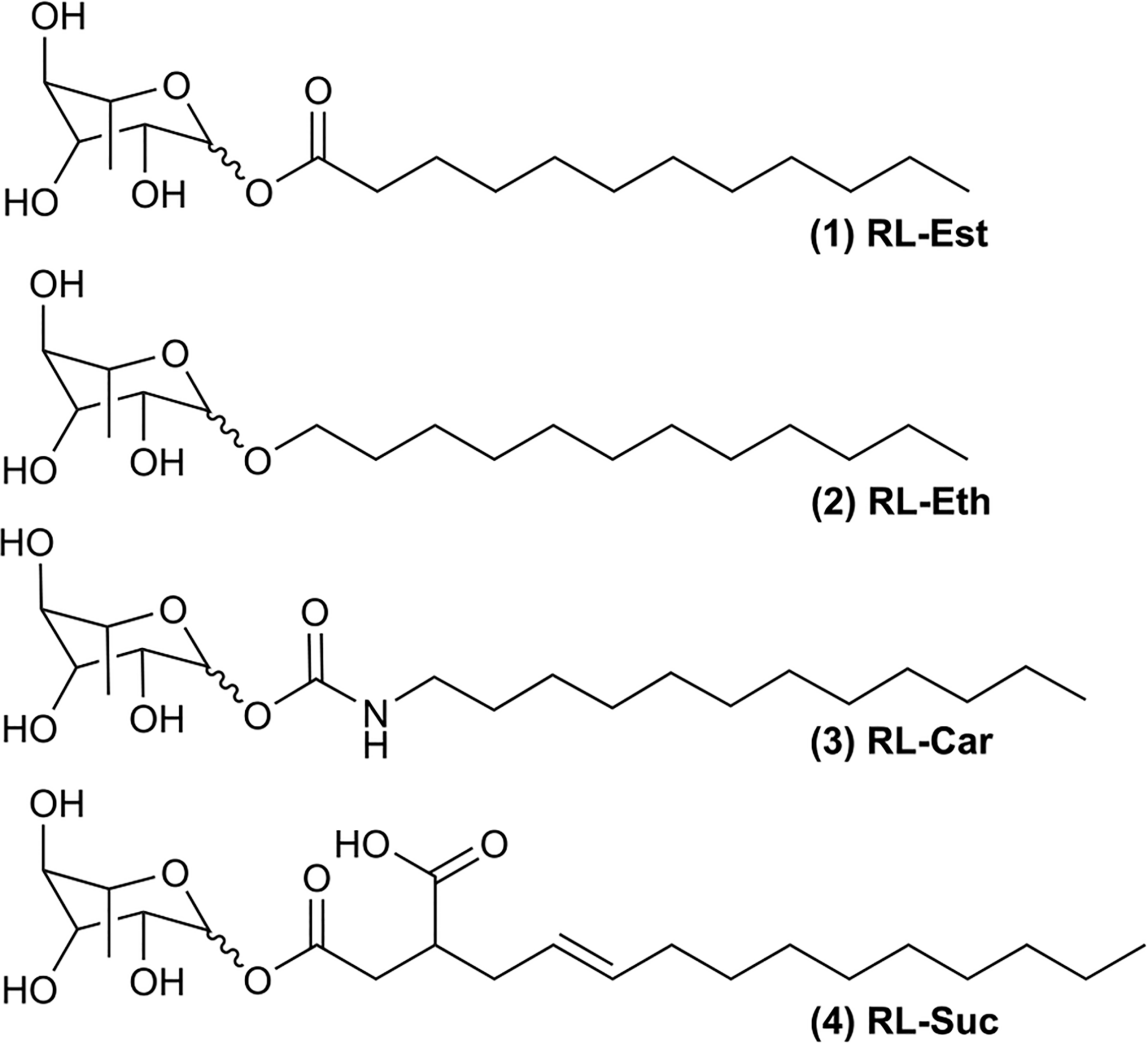
Chemical structures of synthetic rhamnolipids: (1) Rhamnose laurate (RL-Est); (2) Dodecyl rhamnoside (RL-Eth); (3) Rhamnose dodecylcarbamate (RL-Car) and (4) Mono-1-*O*-rhamnosyl (3-dodecenyl) succinate (RL-Suc).

Our long-term research goal is to find novel colloidal systems capable of encapsulating bioactive hydrophobic compounds (e.g.: essential oils) to enhance their performance as plant bioprotectants. Therefore, here we aimed to study the physicochemical and plant-protective properties of four bio-inspired synthetic mono-rhamnolipids with the same acyl length but different sugar-lipid bonds (**Figure 1**). We propose that small chemical variations to the natural rhamnolipids can result in new or enhanced bioactive properties.

The present work reports for the first time the surface activity of synthetic RLs and their capacity to stabilize nanoemulsions. This type of submicron emulsion is a colloidal dispersion system that is long-term kinetically stable under certain experimental conditions [21], in contrast with micro-sized droplet emulsions, which are unstable and tend to coalesce. The developed RLs nanoemulsion formulations were stable for several months and resistant to microbial growth. These nanoemulsions also exhibited the capacity to act as an IR-stimulus, increasing the immune response of the model plant *Arabidopsis thaliana* to a bacterial infection, in an enhanced way in comparison with the RLs suspensions (Robineau et al., 2020). Additionally, synthetic RLs were innocuous to native substrate microbiota and beneficial soil-borne microbes.

## Materials and methods

### RLs sources

Synthetic mono-rhamnolipids were synthesized as described (Robineau et al., 2020). Briefly, the rhamnose ether derivative was obtained from the reaction between L-rhamnose and fatty alcohol with 12 carbon atoms, without solvent, and in the presence of *p*-toluenesulfonic acid as a catalyst with a yield of 42–64%. Acyl derivatives were obtained by esterification of L-rhamnose with dodecanoyl chloride in *N, N*´-Dimethylformamide (DMF) in the presence of *N, N*-Dimethylpyridin-4-amine (DMAP), with a 54% yield. Using similar reaction conditions to obtain an ester derivative, mono-rhamnosyl (dodecenyl) succinate was synthesized using a commercially available (dodecenyl) succinic anhydride. Finally, the carbamate derivative was obtained by a reaction between dodecyl isocyanate and L-rhamnose in a basic medium with a 48% yield. Natural RL extract (purity 90%) from *Pseudomonas aeruginosa*, as well as *n*-hexadecane (HD), salts and buffer reagents, were purchased from SIGMA-ALDRICH (Argentina).

### Spectroscopic analysis of synthetic RLs

NMR analysis of synthetic RLs is shown in the “**Supplementary Material**” file. ^1^H NMR and ^13^C NMR experiments were run using a Bruker Avance II 400 high-resolution spectrometer (^1^H at 400.13 MHz and ^13^C at 100.03 Hz). The solutions for NMR experiments were prepared by dissolving the RL samples in DMSO-d6. Besides, the chemical shifts were reported in parts per million (ppm) and are in agreement with the previous report (Robineau et al., 2020). The residual solvent signal was taken as the reference (Gottlieb et al., 1997).

### Surface activity of RLs suspensions

The surface activity of mono-rhamnolipids was evaluated through surface tension (γ) measurements using the Wilhelmy method, as a function of subphase concentration. Aliquots of 0.5 mL of RLs on 5mM phosphate saline buffer subphase (pH7.4) were tested after 20 min of stabilization in duplicates. The surface activity curves were fitted *via* non-linear least-squares regression analysis as follows (Davies and Rideal, 1963; Zulueta Díaz et al., 2018; Fanani et al., 2022)∶


$$\text{Γ}=\frac{C}{RT}\frac{\text{Δ}\pi }{\text{Δ}C}$$

(Eq. 1), where Γ is the amphiphile surface excess concentration, R is the gas constant, T is the temperature, C is the subphase amphiphile concentration and π is the surface pressure defined as the difference between the surface tension of the bare air/buffer interface (γ_0_) and the γ reached after equilibration with the amphiphile added to the subphase (π=γ_0_-γ, Eq. 2). From the maximum π value, the mean area occupied by a single amphiphile at maximal surface concentration can be calculated as


$$MMA=1/N{\text{Γ}}_{max}$$

(Eq. 3), where N is Avogadro’s constant. All experiments were performed at 22 ± 1°C.

### RL-containing nanoemulsion formation

Oil/water (O/W) nanoemulsions were prepared using *n*-hexadecane (HD) as the model oil phase by the ultrasonication technique (Gurpreet and Singh, 2018). The process involves three steps: I) The pre-emulsification was performed by adding 10 mL of 1 mM RL suspension (in phosphate buffer 5 mM pH 7.4) to the required amount of HD placed in a glass vial under vigorous agitation (1000 rpm for 1h); II) The obtained emulsion was sonicated during 16 cycles (15 s of sonication, 15 s pause) in a Cole Palmer 4710 Series 50 W Ultrasonic Homogenizer with a 3.8 mm diameter tip at 50% of amplitude and III) The sample was stabilized by continuous agitation at 1000 rpm during 24h at room temperature (Multipoint stirrer Froggy6 INGELAB). For plant experiments, HD controls were performed with a mechanical mixture of HD (1% v/v) and buffer, subjected to the pre-emulsification and sonication processes and were used within 1h post sonication.

### Particle analysis

The morphology and size analysis of the oil droplets in the nanoemulsions was performed by transmission electron microscopy (TEM). Nanoemulsions visualization was achieved by adding phosphotungtic acid onto the samples mounted on a gold grid. The TEM micrographs were acquired using a transmission electron microscope (TEM Jeol 1200 EX II) operating at 100kV. The droplets diameter was measured by the analysis of ≥ 210 nanodroplets from 10 images by two independent operators using the open-source Fiji software (http://fiji.sc/Fiji, Biomedical Imaging Group, EPFL) (Schindelin et al., 2012).

Dynamic light scattering (DLS) and ζ potential measurements were used to assess the size distribution and electrostatic properties of oil droplets formed in aqueous solutions. These techniques provide information on the population size distribution of the particles formed in the nanoemulsion and their surface charge at the displacement interface. Likewise, these non-destructive techniques were used to evaluate the colloidal stability of the nanoemulsions as a function of time.

The size distribution of particles was studied by DLS using a 530 nm laser beam, and analyzed at 90° through the adjustment of a correlation function, once a week for 90 days. The equipment used was a Nicomp™ 380 Submicron Particle Sizer (PSS-NICOMP Santa Barbara, California, USA). The hydrodynamic diameter of the particles was determined by the Stokes-Einstein equation. ζ potential measurements were performed using the Zetasizer SZ-100-Z equipment (Horiba, Ltd., Japan), provided with a semiconductor laser excitation solid state (532 nm, 10 mW) and using the laser-Doppler velocimetry technique (Zulueta Díaz et al., 2018). The lipid particles were suspended in 5 mM Tris-HCl, pH 7.4.

### Contact angle analysis

The contact angle of suspensions and nanoemulsions containing the different RLs was determined on the surface of freshly excised *Arabidopsis thaliana* leaves or hydrophobic glass slides. The greater the angle of the water droplet to the surface is, the lower it is its surface energy and, therefore, its adhesion capacity (Davies and Rideal, 1963). For this purpose, leaf-sections without the central vein were attached to a slide using double-sided adhesive tape (Liu et al., 2016). To minimize the gravity effect on the droplet shape (see https://imagej.nih.gov/ij/plugins/contact-angle.html), small droplets (4 μL size) were deposited on the leaf surface and photographed. Hydrophobic glass slides were prepared by alkylation with octadecyltrichlorosilane of acid-clean glass slides in order to self-assemble a covalently linked monolayer of octadecylsilane (von Tscharner and McConnell, 1981) and used similarly to obtain droplets images. The quality of the hydrophobic coverage of each coverslip was checked before use based on the contact angle and free-running of double-distilled water droplets deposited on their surface. Droplets images were taken with a camera with telescopic zoom (Canon SX530 HS). These were processed using the Plugin “Drop analysis – LB-ADSA” (Stalder et al., 2010) from the open-source Fiji software (http://fiji.sc/Fiji, Biomedical Imaging Group, EPFL) (Schindelin et al., 2012) by two independent operators.

### Structural analysis of the RLs

All the molecules were drawn using the software ChemDraw Profesional 16.0 (PerkinElmer Informatics Inc.). Then different descriptors were calculated using the software MarvinSketch 23.4 (ChemAxon Ltd) as follows. The Hydrophilic – Lipophilic Balance (HLB) represents the degree of hydrophilicity or lipophilicity of a molecule (Griffin, 1949), and it was calculated using the Davies method based on the chemical groups of the molecule (Davies, 1957). This considers the effect of stronger and weaker hydrophilic groups and is appropriate for calculating the HLB of ionizable species. On the HLB scale (from 0 to 20), a surfactant molecule with HLB values between 4–8 is more suitable for stabilizing a W/O emulsion, whereas between 8 - 18 are most commonly used for stabilizing O/W emulsions (De et al., 2015). The protonation state (pKa value) of each molecule was calculated based on its partial charge distribution in the static option, which uses the neutral state of the submitted molecule at a temperature of 298°K.

### Plant growth

Arabidopsis (*Arabidopsis thaliana*) Col-0 plants were grown in a commercial substrate (Grow Mix Multipro, Terrafertil SA) at 20-22°C with 60-70% relative humidity and a 12-h-light/12-h-dark cycle (light intensity 150 μE) for 6-8 weeks. Fully expanded leaves were sprayed with different RLs at 300 μM concentration formulated as suspensions in potassium phosphate buffer 5mM pH 7.4 or as nanoemulsions with 1% HD diluted in the same buffer. The buffer and a simulated nanoemulsion with HD but without RLs were used as controls.

### Trypan blue, DAB, and callose stainings

To evaluate plant cell death, leaves pre-treated for 24h with the indicated RLs suspensions or nanoemulsions (300μM) were excised, placed into 15 mL falcon tubes and stained with lactophenol trypan blue staining mix following the standard protocol (Parker et al., 1996). Whole leaves were photographed with a digital camera. Accumulation of ROS was detected by DAB (3,3 diaminobenzidine) staining (Vanacker et al., 2000). For this purpose, 24h after the spray with RLs, leaves were sectioned and dipped in DAB solution (1 mg/ml in water, pH = 4) for 8 h, bleached in 96% ethanol for another 24h, re-hydrated, mounted in glycerol 50% and photographed with a digital camera. These images were transformed to 8-bit depth, inverted and the mean of grey intensity was quantified using Image J software (NIH-USA). Two independent experiments were analysed, with similar results. One-way ANOVA with Duncan’s *post-hoc* test was carried out using InfoStat software (FCA-National University of Cordoba). For callose detection, leaves pre-treated for 24h with the mentioned RLs or controls (negative: 5mM phosphate buffer or HD and positive: *Pseudomonas syringae* DC3000 pv tomato *hrcC-* mutant, (hrcC)) were cut and de-stained in ethanol 96%, re-hydrated in water and dipped in aniline blue solution (0.01% w/v in potassium phosphate buffer 150 mM, pH = 9.5) for 1 hr. Leaves were mounted in 50% glycerol, and images were obtained with an Axioplan 135 microscope (Zeiss, Germany) associated to an Axiovision camera system using UV light illumination and a blue filter with a 20X objective (Fabro et al., 2011). Callose deposition dots were quantitated with the Image J software (NIH-USA) using the Find Maxima function and an *ad-hoc* macro. A non-parametric ANOVA (Kruskal Wallis test) was carried out using InfoStat software. Two independent experiments were analyzed with similar results.

### Bacterial growth curves

Eight-week-old rosettes (adult plants) were sprayed with 300 μM of RLs formulated as suspensions or nanoemulsions (3 mL per 6-plants trays). Three days post-treatment, the plants were inoculated with the hemibiotrophic plant pathogenic bacteria *Pseudomonas syringae* pv tomato DC3000 (Pst-DC3000). For the inoculum, bacteria were cultured overnight at 28°C in liquid LB medium, supplemented with rifampicin (50 mg/mL) and kanamycin (50 mg/mL). Subsequently, bacterial cells were collected by centrifugation and resuspended in 10 mM MgCl_2_ to an optical density (OD at 600 nm) of 0.0005 and inoculated with needle-free syringes into the abaxial surface of RLs-pre-treated *Arabidopsis* leaves. Bacterial growth curves were carried out by collecting 3 sets of 4 leaf discs each from different plants per treatment per time point, grinding in 10 mM MgCl_2_ and plating serial dilutions in LB agar with appropriate antibiotics at day 1 and day 4 post-inoculation. Bacterial colonies were counted after 24h of plates incubation at 28°C, and the results are presented as colony-forming units -CFU-/cm^2^. Bacterial growth curves were performed by duplicate. Statistical analysis was performed by One-Way ANOVA with Dunnett’s *post hoc* test using InfoStat software.

### Evaluation of toxicity to microorganisms present in the substrate

To evaluate the nanoemulsions’ effect on soil microbiota, pots containing commercial substrate (Grow Mix Multipro, Terrafertil SA) were sprayed with 2 mL of 300 µM RLs-stabilized nanoemulsions containing 1% HD. The pots were incubated and maintained under the same conditions as the growing plants. Six days after spraying, 4 samples of 1 g of soil were collected for each condition, and the inoculum of microorganisms was extracted with 9 mL of sterile physiological solution (9% NaCl) by vortexing for 1 min as previously reported (van Overbeek et al., 1995). Samples were decanted, and 100 µL of the supernatants were plated on LB agar plates. The plates were incubated for 24h at 25°C, scanned, and the area covered with microorganisms was calculated by performing a round mask with ImageJ, pre-processing the images as for the DAB staining. Statistical analysis was performed by One-Way ANOVA with Tukey’s *post hoc* test using InfoStat software.

### Evaluation of RLs effect on Pseudomonas fluorescens PF01 and Pseudomonas syringae pv tomato DC3000

To evaluate the nanoemulsions effect on the growth of the beneficial bacterium *Pseudomonas fluorescen*s PF01 (Pf) and the pathogenic bacterium Pst-DC3000, agar diffusion assays were performed. For this purpose, Mueller-Hinton agar plates were inoculated with 100 µL of an inoculum at 0.5 McFarland density (1.5 × 10^8^ CFU/mL). Within 15 min, sterile filter paper discs of 5 mm in diameter were embedded with 10 µL of 300 µM of RLs stabilized nanoemulsions containing 1% HD. Antibiotic-impregnated discs served as a positive control for bacterial growth inhibition, while those embedded in buffer served as the negative control. Plates were incubated for 24h at 28°C and the diameter of the inhibition zone was measured with a Vernier caliper.

### RNA extraction and real-time qRT-PCR

For each sample of plants pre-treated with RLs suspensions or nanoemulsions (300μM), 4 leaves (80 mg) were ground in liquid N_2_ and used to extract total RNA with the hot phenol method (Verwoerd et al., 1989). Then 2 μg of total RNA was used for reverse transcription using M-MLV reverse transcriptase (TransGenBiotech AE101) according to the manufacturer’s instructions. Transcripts expression levels were determined by real-time qRT-PCR using the CFX96 system (BIO-RAD) and a SYBR Green Master Mix qPCR kit as recommended by the manufacturer (GreenLight Master Mix, INBIO Highway). PCR reactions were carried out in triplicate in 8 strip-PCR tubes (10 μL per well) in a buffer containing 5 μL of qPCR master mix, 4 μL of forward and reverse primers (0.5 μM each), and 1 μL of 1/4 dilution of cDNA. After denaturation at 95°C for 15min, amplification occurred in a three-step procedure: 15s of denaturation at 95°C, 15s of annealing at 60°C and 15s of extension at 72°C with a total of 40 cycles. The same cycling conditions were used for all targets. The amplification efficiency (E) on each well was estimated using LnReg software and the mean E for each amplicon was used to determine relative gene expression using Ubiquitin 5 -UBI5- as reference according to the following equation: E^–ΔΔCt^, where ΔΔCt = (Ct GI [unknown sample] – Ct GI [reference sample]) – (Ct UBI 5 [unknown sample] – Ct UBI 5 [reference sample]). GI is the gene of interest. Specific primers were obtained from the literature and are described in **Supplementary Table S1**. Two independent experiments were analysed. Results correspond to means +/- SD of triplicate reactions of one representative experiment. Statistical analysis was performed by One-way ANOVA with Tukey *post hoc* test using InfoStat software.

## Results

### Surface activity of synthetic RLs

The surface activity of the four synthetic mono-rhamnolipids shown in **Figure 1** was studied compared to the natural mixture of RLs produced by *Pseudomonas aeruginosa* (RL-Nat). The effectiveness of a surfactant is determined by its efficiency in reducing surface tension (De et al., 2015; Myers, 2020). This capacity is relevant for evaluating the emulsifying potential of the synthetic RLs. An enormous increase in the interface area is required for a two-phase system to be dispersed in nanometric-size particles of the dispersed (oil) phase. The surface tension counteracts this increase in area. Therefore, a low surface tension will allow an area expansion (and oil phase dispersion) with a lower energetic cost.

When RLs compounds were added to the bulk of a saline solution they self-aggregate into a monolayer at the air/water interface resulting in a “Gibbs monolayer” (**Supplementary Figure S1**) (Fanani et al., 2022). A decrease in surface tension (γ) occurs according to the Gibbs adsorption equation (Eq. 1) (Davies and Rideal, 1963). The activity of a surfactant depends on its concentration until the critical micelle concentration (CMC) is reached. Measuring the γ of different concentrations of each RL solution, the CMC was obtained as the highest concentration that obeys the Gibbs adsorption equation (Eq.3 in section 2.3), meaning that the amphiphiles are present as monomers (Fanani et al., 2022). Above this concentration, amphiphiles form nanostructures, possibly micelles or vesicles.

The minimum γ reached at the CMC as well as the CMC values are shown in **Supplementary Figure S1** and **Table 1**. For RL-Nat suspensions a CMC of 0.2 mM was observed, which agrees with previous reports (Mańko et al., 2014; Bai and McClements, 2016). In accordance, Ozdemir and co-workers reported CMC values at neutral pH of 0.1 and 0.15 mM for mono- and di-RL, respectively (Özdemir et al., 2004). Synthetic RL suspensions had CMC values in the 0.05 mM to 0.5 mM range, with the maximum value for RL-Car and the minimum for RL-Suc. At the CMC, the minimum γ reached for the RL-Nat suspension is 30 mN/m, in agreement with (Özdemir et al., 2004; Mańko et al., 2014; Liu et al., 2016). The synthetic RLs showed similar or lower values, being RL-Suc more effective.

**Table 1:** Summary of RLs molecular parameters obtained from surface activity experiments and molecular structural analysis.

RL	MW* (g/mol)	γ_min_ (mN/m)	CMC (mM)	MMA_CMC_ (Å^2^/molec)	Γ (mol/m^2^)	HLB*	pKa *							
							pK 1		pK 2		pK 3		pK 4	
RL-Nat	650	30 ± 2	0.2 ± 0.1	91 ± 28	(20 ± 7)x10^-7^										
RL-Est	346.5	28.5 ± 0.1	0.2 ± 0.1	56 ± 11	(30 ± 6)x10^-7^	8.33	12.2		14.8		13.3			
RL-Eth	332.5	28 ± 1	0.04 ± 0.01	47 ± 1	(35 ± 1)x10^-7^	6.75	12.2		14.8		13.3			
RL-Car	375.5	30 ± 3	0.5 ± 0.1	59 ± 4	(28 ± 2)x10^-7^	7.85	12.2		14.7		13.3		15.6	
RL-Suc	430.5	26.7 ± 0.3	0.05 ± 0.01	28 ± 2	(60 ± 5)x10^-7^	9.95	12.2		14.8		12.2		4.2	

*These parameters were calculated by molecular structural analysis as stated in Section 2.7, MW in agreement with (Platel et al., 2021) and average Rh-Nat MW was taken from (Jarvis and Johnson, 1949).

Regression analysis of the γ measurements by adjusting the Gibbs equation gives access to surface concentration (Γ) and the mean area occupied by the RL at the saturated surface (MMA). **Supplementary Figure S1** and **Table 1** show that the RL-Nat occupies the larger MMA. This result is in the range reported for mono- and di-RL (Özdemir et al., 2004), which is highly dependent on the pH (59-135 Å^2^/molecule). Synthetic RLs showed MMA values between 47 and 59 Å^2^/molecule, an expected area for a typical lipid at the interface (Gaines, 1966), except for RL-Suc, which showed a MMA of 28 Å^2^/molecule evidencing a more compact surface packing. Being the RL-Nat a complex mixture of amphiphiles that usually present more than one hydrophobic tail, the MMA is expected to be larger than the synthetic mono-RLs found.

A close analysis of **Table 1** shows that RL-Suc is the more effective surfactant, since the lowest minimum γ (26.7 mN/m) is reached by a low concentration (only 0.05 mM) of monomeric amphiphiles, followed by RL-Est, which reduces the γ to 28 mN/m at CMC concentration (0.04 mM). As a comparison, sodium dodecyl sulfate (SDS), a powerful industrial surfactant shows a CMC of 8-9.4 mM (Khan and Shah, 2008) and a minimum γ of 28 mN/m (De et al., 2015). On the other hand, all the synthetic RLs studied showed low stability at the air/water interface. They did not form Langmuir films, since we observed a decrease in the area occupied by monolayers of RLs spread onto a saline solution at pH 7.4 registered at a constant π (Eq. 2) of 20 mN/m as a function of time (not shown).

Hydrophilic-lipophilic balance (HLB) value provides a prediction of the surfactant property and is a measure to indicate the type of emulsion able to stabilize. The HLB value varies between 0 and 20 and, as a general rule, water-soluble surfactants (high HLB) yield oil–in–water (O/W) systems while oil-soluble materials (low HLB) preferentially produce water-in-oil (W/O) emulsions (De et al., 2015; Myers, 2020). In the present work, HLB values for the synthetic RLs were calculated using the tool HLB predictor from MarvinSketch 23.4 (ChemAxon Ltd). The HLB found for the synthetic RLs ranges between 6.75 – 9.95, which predicts a good O/W emulsifier power for RL-Suc (9.95) and RL-Est (8.33). In contrast, the values obtained for RL-Car (7.85) and RL-Eth (6.75) indicates that these are molecules more suitable for stabilizing W/O emulsions (De et al., 2015; Myers, 2020).

Finally, the acidity of the oxygen atoms constituting the synthetic RLs was also calculated using the same software at neutral pH. **Table 1** indicates that all the ring oxygens (see **Figure 1**) have very high pK and only the carboxylic oxygen of RL-Suc shows a pK below neutrality. It is worth noticing that this behavior is expected to be found in monomeric amphiphiles. When the RL-Suc were organized at a surface, an ion double layer effect should be expected and the effective pK observed should be shifted to higher bulk pH (Benedini et al., 2011; Galassi and Wilke, 2021).

### RL-stabilized nanoemulsions

Colloidal systems with droplets size ≥ 50 nm are thermodynamically unstable and tend to coalesce or incur into Ostwald ripening over time (McClements, 2012). To hinder the destabilization and subsequent breaking of the emulsion, the use of potent amphiphiles and preferably with a net charge providing either electrostatic or steric stabilization to the droplets is necessary, as well as, the use of a hydrophobic oil that can counteract the droplet’s osmotic pressure. In the present work, HD was chosen as the model oil phase due to its remarkably low solubility in water, which increases the droplets’ stabilization and effectiveness (Landfester, 2006). RL-Nat has been described as having excellent emulsifying power (Lovaglio et al., 2011). Furthermore, several works reported the formulation of RL-Nat-based nanoemulsions (Bai and McClements, 2016; Khan et al., 2022) particularly for their use in crude oil recovery (Onaizi, 2021; Al-Sakkaf and Onaizi, 2022). Here we report the formulation of natural as well as synthetic RL-stabilized nanoemulsions.

**Figure 2:**
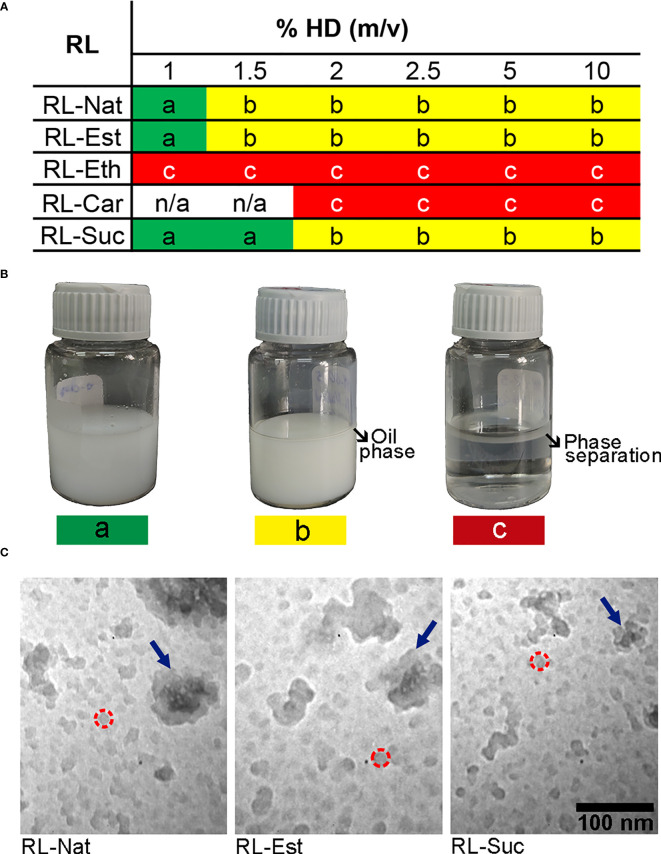
Summary of the rhamnolipid O/W emulsions outcome prepared with different oil/water volume ratio. **(A)** The nanoemulsions prepared with 1mM RL suspension and different RL and %HD were assessed and classified (color code) after one week of storage at room temperature without stirring as following: a) stable emulsion (green), b) partial emulsification (yellow), c) phase separation (red). **(B)** Images of representative nanoemulsions preparations showing the colloidal stability related to the color code classification. **(C)** TEM images of nanoemulsions containing RL-Nat (left), RL-Est (center) or RL-Suc (right). Representative nanodroplets are circumscribed by a dashed red circle and the nanodroplet`s aggregates are highlighted with blue arrows.

**Figures 2A, B** show the outcome of nanoemulsion formulation using 1 mM RL suspension and varying the oil phase proportion (%HD). All the RLs were above 2 x CMC values at this concentration, and therefore their monomer concentration is the maximum obtained. RL-Nat, RL-Est, and RL-Eth emulsified 1% of the oil phase, while RL-Suc successfully emulsified 1.5% of HD. Above this oil content, nanoemulsions were found in equilibrium with the HD phase. However, the RL-Eth nanoemulsion incurred phase separation after one week of storage. On the other hand, RL-Car failed to stabilize the nanoemulsions with HD at all the studied conditions. The results obtained for RL-Eth and RL-Car emulsions confirm that they are not good stabilizing agents for O/W emulsions, which agrees with the calculated HLB values. Based on these results, we selected the 1% RL-Nat, 1% RL-Est and 1.5% RL-Suc nanoemulsions for further investigation.

The nanoemulsions droplets size distribution was analysed by transmission electron microscopy (TEM) and Dynamic Light Scattering (DLS). **Figure 2C** clearly show the occurrence of very small (11 - 28 nm) nanodroplets in the RL-Nat, RL-Est and RL-Suc-stabilized nanoemulsions with similar but significantly different main diameter (**Table 2**) and larger aggregates of such particles. The thermodynamic stability of colloidal systems has been proposed to depend on the droplet size (McClements, 2012). In this theoretical approach, the surface tension shows a minimum when the droplet surface curvature coincides with the spontaneous curvature of the surfactant. In such condition, the entropic contribution predominates achieving thermodynamic stability. For the systems studied this is reached at droplets sizes close to 15 nm, strongly depending on the surfactant and phases composition (McClements, 2012).

**Table 2:** Physicochemical characterization of the selected rhamnolipids’ nanoemulsions.

RL	HD RH	% HD (m/v)	TEM Diameter (nm)*	Diameter** (nm)	PDI	ζ-potential** (mV)	Emulsion Stability (days)
RL-Nat	45:1	1	19 ± 6	240 ± 24	0.97 ± 0.67	-55 ± 2	64
RL-Est	45:1	1	23 ± 5	231 ± 48	0.23 ± 0.10	-40 ± 10	>92
RL-Suc	68:1	1.5	16 ± 5	283 ± 53	0.24 ± 0.12	-0.12 ± 0.4	>92

*Average of n ≥ 210 particles. The differences between the three samples are statistically significant with p-value< 0.01.

**ζ potential values are the average of n=20 and diameter of n=28 samples ± SD. PDI was calculated as the square of the standard deviation divided by the mean particle diameter.

DLS analysis of nanoelmulsions showed a predominance of larger particles (220 – 300 nm), which probably reflects the occurrence of the droplet aggregates observed by TEM (**Table 2**). However, our experimental setup fails in detecting the 11-28 nm size particles, from which they are formed. TEM is a number-based particle size measurement whereas DLS is an intensity-based one. Therefore, DLS is very sensitive to large particles, whilst TEM shows a stronger emphasis on the smallest components in the size distribution (Klang et al., 2012).

The droplet size of nanoemulsions stabilized by RL-Nat has been shown to depend on the oil phase used. Bai and coworkers reported an average droplet size of ~110 nm determined by DLS for medium chain triglycerides and ~500 nm for lemon oil after 7 days of storage, while ζ potential values were in the -60 to -70 range at pH6 (Bai and McClements, 2016). On the other hand, nanoemulsions of RL-Nat using long-chain glycerides as oil phase resulted in droplet diameters ranging from 21 to 336 nm (Polydispersity Index (PDI) ranged from 0.178 to 0.65) and ζ potential values ranged from −29.6 to −55.3 mV (Khan et al., 2022).

Our results for the RL-Nat nanoemulsion broadly agree with those reports. However, the PDI analysis evidences a very polydisperse colloidal preparation (see classification in (Osmani et al., 2014) and **Table 2**), in agreement with a higher frequency of occurrence of a second (and larger) droplet population. DLS analysis of nanoemulsions stabilized by RL-Est and RL-Suc showed droplet size in the 200-300 nm diameter and lower PDI, evidencing a mid-range polydispersity (Osmani et al., 2014). RL-Est-containing nanoemulsions showed a ζ potential value close to the values observed for RL-Nat. Being this amphiphile neutral at pH 7.4, the negative value of ζ potential observed may respond to surface adsorption of ions, as is the case of surfaces formed by the zwitterionic lipid phosphatidylcholine (Zulueta Díaz et al., 2018).

On the other hand, RL-Suc showed values close to neutrality. Considering that the later RL shows a carboxylic group with a proposed pH of 4.2 (**Table 1**) this result is, at least, unexpected. A comparison with the ionization surface behavior of ascorbyl palmitate (ASC16), an amphiphilic compound with an acidic hydroxyl (pK 4.2) might bring light to this phenomenon. ASC16 showed a surface behaviour sensitive to both pH and ionic strength. Using a theoretical approach, we could explain this behaviour based on a surface pH decrease of two units at bulk pH 6 in high salt concentration and a surface packing corresponding to 30 Å^2^.molec^-1^. This effect results in a partial neutralization of the amphiphiles with a dissociation fraction of 0.4. Since this effect is a consequence of the establishment of an ionic double layer (Davies and Rideal, 1963), it is exacerbated at low ionic strength conditions (Benedini et al., 2011).

At the surface of RL-Suc-stabilized droplets, a monolayer of surfactant is self-organized to form a charged surface at neutral pH, attracting proton ions and lowering the surface pH. If the surface pH is reduced near the RL-Suc pK (4.2), this will affect the ionization of the amphiphile neutralizing the surface. However, the ionic double layer should remain to alter the surface electrostatically. According to the theoretical calculations in (Benedini et al., 2011), in the conditions used in our experiments, a lowering of the RL-Suc surface pH of ~3 units from 7.4 to ~4.4 can be expected, thus partially neutralizing the surface charge and explaining a ζ potential value near neutrality.

The droplet size distribution of the selected nanoemulsions determined by DLS was analyzed as a function of time at room temperature without stirring. All the samples had hydrodynamic diameters between 230-290 nm (**Table 2**) and were colloidal stable for at least 3 months as is shown in **Supplementary Figure S2**, except for the 1% RL-Nat sample. The measurements were taken once per week for two independent samples. It is important to note that the samples showed the presence of a second population of larger diameter (1-2 µm), which appeared in a frequency of 0.4, 0.3, and 0.2 for 1% RL-Nat, 1% RL-Est, and 1.5% RL-Suc, respectively. Since the frequency of appearance during the 3 months of storage was lower than 0.5, they were not included in the analysis.

It is worth noticing that RL-Est and RL-Suc samples did not show signs of microbial growth during the storage period under study even when no bacteriostatic agent or sterile conditions were used for their preparation. RL-Nat is known to have direct antimicrobial activities against a wide variety of microorganisms (Haba et al., 2003; Vatsa et al., 2010; Araujo et al., 2022), highlighting its potential use in agricultural and biomedical fields. Recently, the RL-Est was described to inhibit the growth of the tomato plant pathogen *Botrytis Cinerea* (Robineau et al., 2020) and the wheat pathogen *Zymoseptoria tritici* (Platel et al., 2021), supporting the potential use of this compound in the agricultural industry.

### Wettability of RL suspensions and nanoemulsions on the plant leaf surface

The RL-stabilized nanoemulsions described here may have applications in a wide variety of industrial areas. Our present interest is focussed on its use in the agroindustry. In this regard, the developed RL-based nanoemulsions are capable of carrying in their oil-phase lipophilic bioactive compounds. This action might be reinforced by the capacity of the studied RL to activate the plant’s innate immune system, contributing to a green alternative for attaining crop protection.

To this purpose, the wettability of the sprayed formulation droplets on the plant leaf surface is a relevant factor that may contribute to, or counteract, the overall biological efficiency of the nanoemulsion, conditioning droplets resisting deposition/evaporation, enhancing penetration across the cuticular membrane and compounds translocation. The cuticle, a wavy layer, which covers plant leaves and protects the plant from the external environment as well as the surface microstructure are important factors for the leaf surface energy and hydrophobicity (Liu et al., 2016; Soffe et al., 2019).

**Figure 3:**
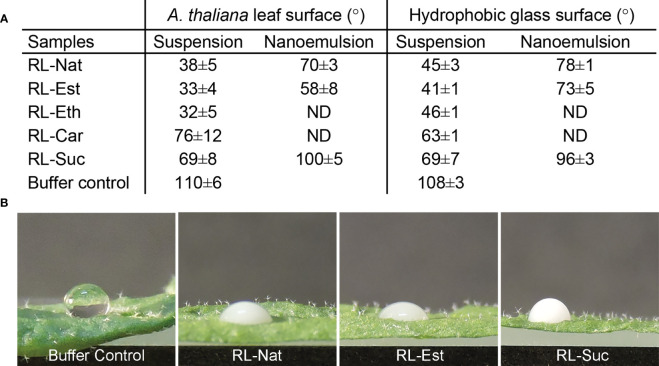
Wettability of RLs suspensions and nanoemulsions. **(A)** Contact angle measurement of 1 mM RLs suspensions and nanoemulsions onto an *A thaliana* leaf or hydrophobic glass surfaces. The values are mean ± SD from two independent samples (in duplicate droplets), analyzed by two independent operators; **(B)** Representative images of the 4μL size drops used to measure the calculated values shown in A.

*Arabidopsis thaliana* is widely used model system for plant genetics, molecular plant biology, and phytopathology. Surfaces can be classified as either hydrophilic, hydrophobic, or superhydrophobic, when the contact angle of water is<90°, >90°, and >150°, respectively. The surface of the leaves of *A. thaliana* has been described as highly hydrophobic, showing a contact angle of 97 ± 1°C (Soffe et al., 2019), a measurement that can be affected by the plant growth condition and the size of the droplet analyzed. Our results showed a water contact angle of 110°C on freshly detached *A. thaliana* leaves. This value remains in the “hydrophobic” range classification. The value reported by Soffe and coworkers (Soffe et al., 2019) may be affected by the gravity effect of the 60 μL size droplet analyzed, an artifact that is minimized in our 4 μL droplets (**Figure 3**).

RL-Nat suspensions have been described to have high wettability on hydrophobic surfaces (Zdziennicka and Jańczuk, 2018) and to show foliar penetration enhancement activity (Liu et al., 2016). A leaf can be accepted as “wettable” if the water contact angle is >90°C and considered “non-wettable” if the water contact angle is <90°C. RL-Nat is able to drop the contact angle of *Iris pseudacorus* leaf surface in 65°C, from 125°C to ~60°C (Liu et al., 2016) and from 110°C to 50°C (a drop of 60°C) on PTFE surface (Zdziennicka and Jańczuk, 2018). We found that RL-Nat suspension lowers the contact angle on *A. thaliana* leaf from 110°C (control buffer) to ~38°C, which results in a 72°C drop (**Figure 3**).

All the synthetic RL suspensions showed high wettability properties on the leaf surface, being RL-Est and RL-Eth more efficient, while RL-Car and RL-Suc were less efficient than RL-Nat (**Figure 3**). We further tested the wettability of the stable RL nanoemulsions obtained. RL-Nat and RL-Est nanoemulsion showed low contact angles, however higher than the corresponding suspensions. On the contrary, RL-Suc nanoemulsion showed a contact angle value >90°C, evidencing a not-wettable behaviour (**Figure 3**).

The wettability of RL suspensions and nanoemulsions was also tested on a planar hydrophobic surface. Modestly higher contact angle values were observed for the RL-Nat, RL-Est, and RL-Eth samples when placed onto an alkylated glass surface, while RL-Car and RL-Suc showed similar or lower values (**Figure 3**). Since the hydrophobicity of the leaf surface account not only for to the surface properties of the wax cuticle but also on its microstructure, comparing the wettability properties to a planar hydrophobic surface might help differentiate both contributions. Our results indicate a minor but significant contribution of the leaf microstructure to the wettability of the RL-Nat, RL-Est and RL-Eth suspensions, which induced a decreasing of the contact angle to<40°C. Furthermore, since RL suspensions and nanoemulsions contain the same RL concentration, the difference in contact angle observed for those corresponding samples may be attributed to a certain amount of HD dissolved into the aqueous phase and affecting the surface property of the nanoemulsions.

### RL nanoemulsions do not trigger plant cell death and neither increase ROS production, or callose deposition

It has been previously reported that RL-Nat, sprayed over plants as suspensions, do trigger an immune response in *A. thaliana* that protects the plant against the attack of pathogens from different kingdoms of life (bacteria, oomycetes, fungi) and with different lifestyles (biotrophs, hemi-biotrophs, necrotrophs) (Sanchez et al., 2012). Here we evaluated if natural and synthetic RLs formulated as nanoemulsions kept or enhanced their plant defense inducers characteristics.

We first tested if nanoemulsions of natural and synthetic RLs at the concentration of 300μM (about 0.2 mg/mL) could cause deleterious effects over plant tissues. For this, we sprayed adult *A. thaliana* plants with three types of RLs (RL-Nat, RL-Est and RL-Suc), formulated either as suspensions or as nanoemulsions. RL-Car and RL-Eth were not tested because they could not form stable nanoemulsions. Leaf samples were collected 24h after treatment and stained to detect cell death with trypan blue (Parker et al., 1996). We did not observe dead cells in any of the RLs treatments either as suspensions or nanoemulsions (data not shown). This is consistent with the observations of Sanchez et al.,when using 0,2 mg/mL of RL-Nat (Sanchez et al., 2012).

**Figure 4:**
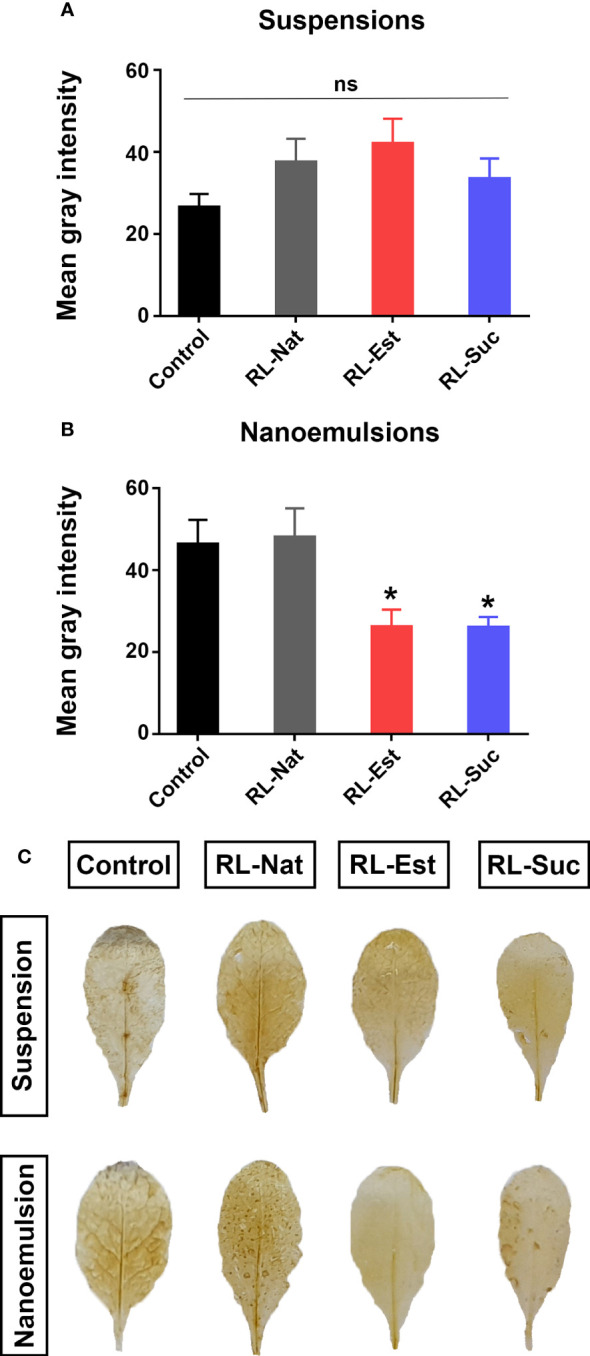
Quantitation of H_2_O_2_ production by DAB staining in *A thaliana* leaves 24 h post-spraying with 300μM RLs suspensions **(A)** or nanoemulsions **(B)**. Asterisks indicate statistical differences according to One-way ANOVA with p<0.05 Duncan’s *post hoc* test. Representative leaves are shown in **(C)**. Control treatments are potassium phosphate buffer for suspensions and HD for nanoemulsions.

To evaluate a broad-spectrum plant defense response as is the production of reactive oxygen species (ROS), the same treatment above described was performed and after 24h, the leaves where stained with DAB (3,3 diaminobenzidine) to detect hydrogen peroxide. As shown in **Figure 4**, the different types of RLs, either as nanoemulsions or suspensions, did not increase ROS production compared with the controls. On the contrary, synthetic RLs applied as nanoemulsions generated even less ROS than the HD control treatment. This is an interesting characteristic, since the generation of large amounts of ROS in plant tissues has been reported as harmful to the plant (Robineau et al., 2020).

**Figure 5:**
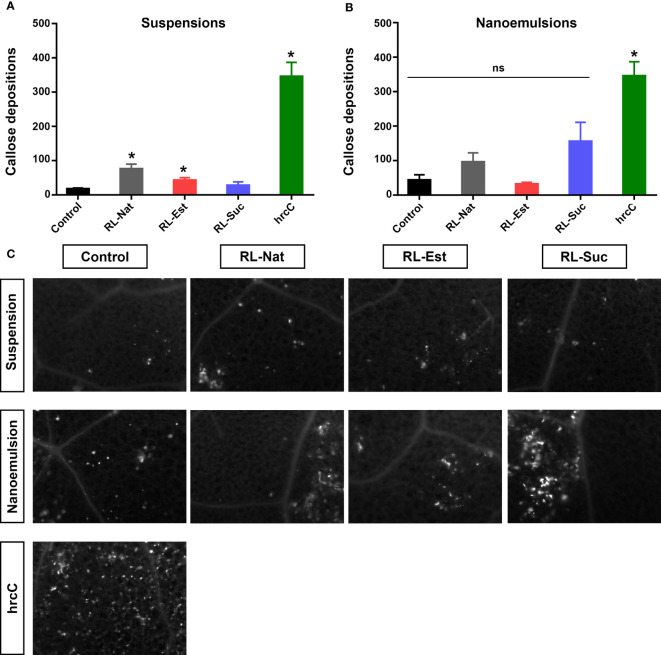
Quantitation of callose deposition dots at the cell wall of *A thaliana* leaves 24h post-spraying with 300μM RLs suspensions **(A)** or nanoemulsions **(B)**. Asterisks indicate statistical differences in a non-parametric ANOVA (Kruskal Wallis) with p<0.05. Representative pictures of callose deposits in the treated leaves are shown in **(C)**. Control treatments are potassium phosphate buffer for suspensions and HD for nanoemulsions. ns, non-significant.

We next tested if the different RLs could trigger the deposition of the (1,3)-β-glucan cell wall polymer callose, which generates cell wall thickenings at sites of microbial attack or mechanical damage, indicatives of active plant defense responses (Ellinger and Voigt, 2014). As positive control we used an inoculation with a mutant of the bacterial pathogen *Pseudomonas syringae* pv. tomato DC3000, the *Hrc-C* mutant (hrcC), that induces a massive accumulation of callose at the plant cell wall. Quantitation of callose deposition after spraying with RLs as suspensions indicated that only RL-Nat and RL-Est generated a slightly higher number of callose dots than the buffer control (**Figures 5A, C**). Regarding nanoemulsions, RL-Suc generated a higher number of callose dots than those induced by its suspension but still this number was not significantly different from the HD control (**Figures 5B, C**). None of the RLs suspensions or nanoemulsions reached the elevated levels of callose deposition triggered by hrcC.

### Pre-treatment with RL nanoemulsions effectively reduces the growth of pathogenic bacteria in planta but not *in vitro*

Previous work (Sanchez et al., 2012) reported that a pre-treatment of *A. thaliana* leaves with RL-Nat at a concentration of 0.2 and 1 mg/mL, 96h before dipping the plants in a bacterial suspension of *Pseudomonas syringae* pv tomato DC3000 (Pst-DC3000), reduced the capability of the pathogen to grow *in planta* but did not affect Pst-DC3000 growth *in vitro*. To test if the synthetic RLs or the formulation of RL-Nat as nanoemulsion had a direct bacteriostatic or bactericidal effect, we exposed Pst-DC3000 to 300μM of the different RLs formulated as nanoemulsions (RL-Nat, RL-Est and RL-Suc). Our results indicate that none of the tested RLs reduced bacterial growth *in vitro* (**Supplementary Figure S3**).

**Figure 6:**
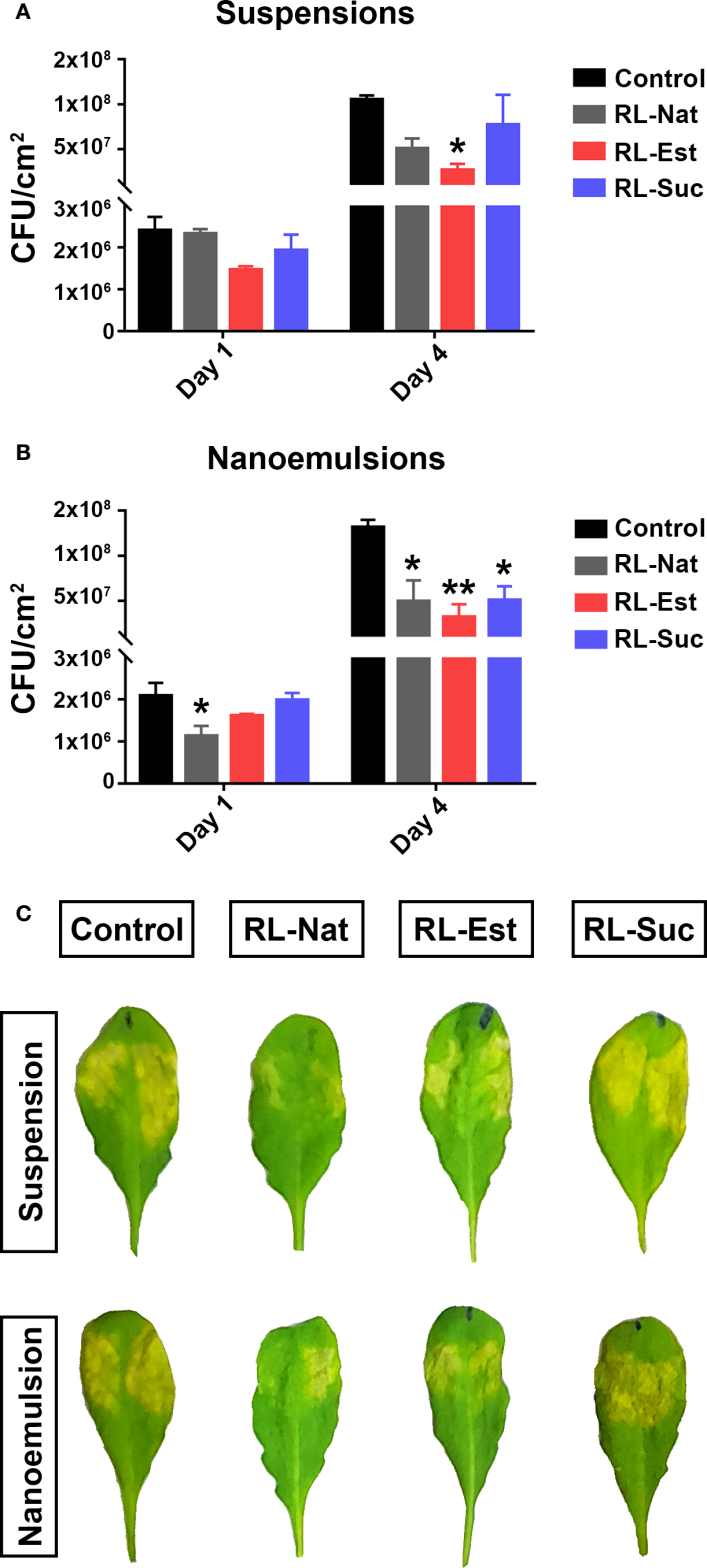
Pre-treatment of *A thaliana* leaves with RLs nanoemulsions protects them against Pst-DC3000 infection. Plants were sprayed with RLs at 300μM formulated either as suspensions **(A)** or nanoemulsions **(B)**. Phosphate buffer or HD were used as controls, respectively. Three days later, leaves were syringe-infiltrated with Pst-DC3000 and bacterial growth was determined at day 1 and day 4 post infection -dpi-. Asterisks indicate significant differences between the RL-treated sample and their control according to One-Way ANOVA with Dunnett’s *post hoc* test (*p<0.05, **p<0.01). Values shown are means +/- SE (n= 12 leaf discs per point) from one representative experiment. Representative images of symptoms observed in infected leaves at 4 dpi are shown in **(C)**.

Then, we studied the effect of RLs nanoemulsions *in planta* by performing bacterial growth curves to determine the level of pathogen infection in leaf tissues. For this, we pre-treated *A. thaliana* Col-0 adult plants by spraying them with the different RLs suspensions or nanoemulsions. Three days later, we syringe-inoculated the leaves with Pst-DC3000 and determined bacterial titers at days 1 and 4 after inoculation. As it can be observed in **Figure 6A**, we could reproduce the previous data by observing a tendency indicating that suspensions of RL-Nat reduced bacterial growth *in planta* (Sanchez et al., 2012). This was also observed for the pre-treatment with RL-Est but not for RL-Suc suspensions, even when there was a reduction in bacterial number, it was not significant. Regarding nanoemulsions, we observed that all the RLs tested caused a significant decrease in bacterial growth, with RL-Est causing the greatest reduction (**Figure 6B**). We also noticed that the disease symptoms visible in leaves did not always correlate with the recorded restriction in bacterial growth, as can be observed in **Figure 6C**.

### Plant defense gene expression in response to RLs

**Figure 7:**
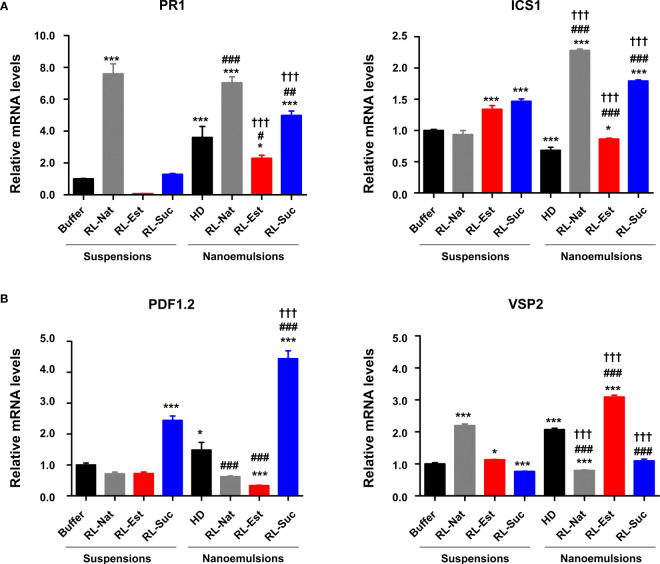
Defense genes expression upon pre-treatment of *A thaliana* leaves with 300μM RLs suspensions or nanoemulsions. **(A)** Expression pattern of SA-marker genes PR1 and ICS1 **(B)** Expression pattern of JA-marker genes PDF1.2 and VSP2. One-Way analysis of variance and Tukey’s Multiple Comparison *post-hoc* Test were applied. (*) indicate significant differences comparing all samples (suspensions and nanoemulsions) versus the buffer control. (#) indicate significant differences between the nanoemulsions and the HD control. (†) indicate significant differences for each RL between its two formulations. Values shown are means +/- SD (n= 12 leaf discs per point) from one representative experiment that was replicated twice. (^x^p<0.05, ^xx^p< 0.01, ^xxx^p<0.001).

To investigate the capability of RLs nanoemulsions to induce plant defense responses at the molecular level, we monitored the expression of four marker genes, two corresponding to the salicylic acid (SA)-signaling pathway (PR1 and ICS1) and another two from the jasmonic acid (JA)-signaling pathway (PDF1.2 and VSP2). The SA pathway is related to the sector of plant defenses that is effective against biotrophic and hemibiotrophic pathogens while the JA pathway activates against necrotrophs and wounding by insects’ attacks (Vos et al., 2015). We observed significant changes in the expression of PR1 at 24hs post application of RL-Nat, either as suspensions or nanoemulsions. Regarding the synthetic RLs, both RL-Est and RL-Suc nanoemulsions increased PR1 expression levels compared to their formulation as suspensions. However, in the case of RL-Est, this change was not higher than the one caused by the HD control (**Figure 7A**, left panel). For ICS1, both synthetic RLs were able to significantly induce this gene expression either as suspensions or nanoemulsions, while RL-Nat was only effective as nanoemulsion (**Figure 7A**, right panel). For the JA-pathway marker genes, we observed that PDF1.2 was significantly induced only by the synthetic RL-Suc suspension and nanoemulsion (**Figure 7B**, left panel). In the case of VSP2, only the RL-Est was effective in both formulations to induce this marker gene, while for RL-Nat, only the suspension induced this gene. On the contrary, neither formulation of RL-Suc induced VSP2 (**Figure 7B**, right panel). Our results indicate that both defense signaling pathways, SA and JA, are induced by RLs, but to a different extent depending on the nature and formulation of each RL here analyzed.

### RL nanoemulsions do not negatively affect substrate-microbiota growth or beneficial soil bacteria

**Figure 8:**
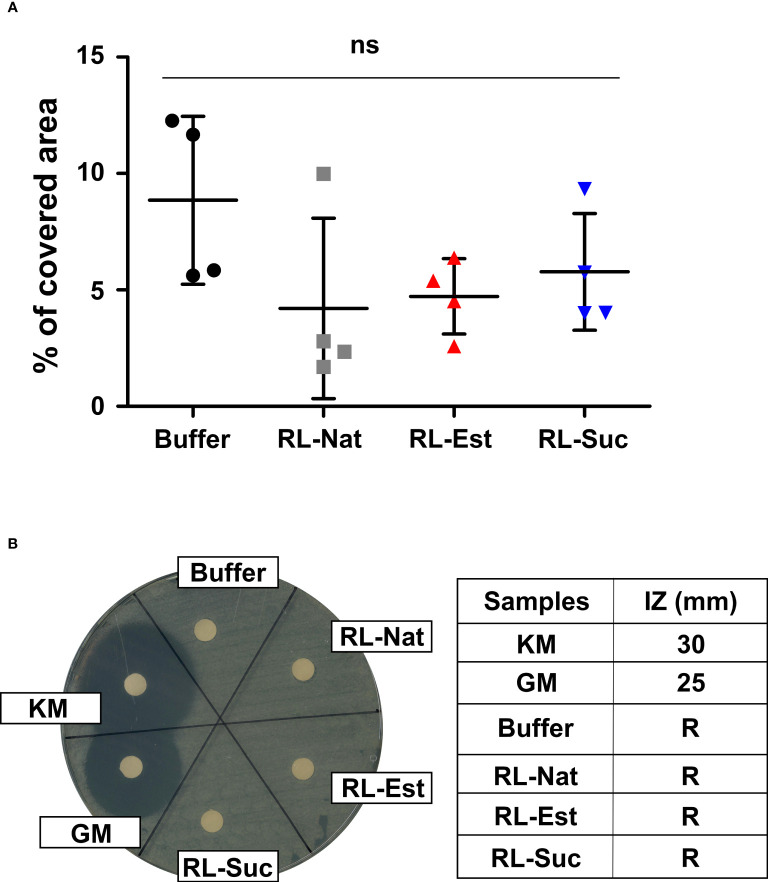
Effect of RL nanoemulsions (300μM) on substrate microbiota and beneficial soil bacteria. Effect of RL nanoemulsions on the growth of **(A)** natural soil microbiota and **(B)** *Pseudomonas fluorescens*. One-way analysis of variance and Tukey’s Multiple Comparison *post-hoc* Test were applied in **(A)**. ns: not significant. **(B)** The halo of the bacterial growth inhibition zone (left panel) and the inhibition zone (IZ) in mm (right panel) are shown. Known antibiotics were used as positive controls: KM (kanamycin), GM (gentamycin). R: resistant.

To investigate if the amounts of RLs sprayed over the plants might negatively affect the survival of natural microbiota present in the plant´s substrate, the nanoemulsions were applied directly by spraying the substrate surface. Six days later, soil samples were taken and re-suspended in sterile physiological solution in order to extract the microorganisms present, which were then plated in rich media and their growth was recorded at 24h. The plate area covered by microorganisms was photographed, measured and compared among treatments. As it can be observed in **Figure 8A**, there is no statistically significant negative effect of the RLs nanoemulsions on natural soil microbiota.

Additionally, we tested if the concentrations of RLs we used could affect the growth of beneficial bacteria, as it is *Pseudomonas fluorescens*, a species usually included in the formulation of bioinoculants used to promote plant health (Vassilev et al., 2020). For this, we performed growth inhibition assays in solid media. As it can be observed in **Figure 8B** the growth of *P. fluorescens in vitro* is not affected neither by the synthetic or natural RLs nanoemulsions used here.

## Discussion

Our laboratories are interested in developing novel bio-carriers for plant protective hydrophobic compounds (e.g. insecticides/deterrents). Thus, we decided to assess the capability of bioinspired synthetic mono-RLs (Robineau et al., 2020; Platel et al., 2021) to generate stable nanoemulsions that besides functioning as such carriers, might have enhanced bioprotective activity *per se*. Here, for the first time, we reported these synthetic mono-RLs’ surface activity and emulsifying power. Notably, all the synthetic RLs equal or surpass the surfactant capacity of the natural mixture, even though they represent a minimal chemical unit of the complex molecules present in the RL-Nat. Both, RL-Eth and RL-Suc stands out from the rest lowering the γ in a similar way than the rest but with a CMC (and a maximal concentration of monomers) an order of magnitude lower.

Chemical structure predictions indicated that RL-Suc and RL-Est had the potential to stabilize O/W emulsions, while RL-Eth and RL-Car would better stabilize W/O emulsions. This was confirmed by the stability studies of O/W nanoemulsions containing HD as the model oil phase. From all the synthetic RLs, RL-Suc appeared as the most powerful stabilizer, in accordance with its ionization capacity and high surface activity. The nanoemulsions with RL-Est and RL-Suc were more stable than those with RL-Nat, showing no sight of microbial contamination or drop coalescence for over 90 days. The small size of the nanodroplets (16 - 23 nm) observed by TEM might be a predominant factor for the high stability of the nanoemulsions.

For their use in plant health maintenance, these nanoemulsions should maximize wettability, thus extending the contact between the product and the plant surface to prolong their bioactivity. We found that RL-Est and RL-Eth suspensions provided better wettability to A*. thaliana* leaf surface than RL-Nat, while the contrary occurred with RL-Car and RL-Suc. Nanoemulsions showed less wettability power than suspensions, standing out the RL-Est-stabilized nanoemulsion, which showed a lower contact angle than RL-Nat (Soffe et al., 2019). From these results, RL-Est appeared as the most promising nanoemulsion stabilizer due to its high stability and wettability. Further functional studies *in planta* confirmed this prediction and showed interesting differences between the capability of the synthetic RL-Est and RL-Suc, compared with the RL-Nat, in providing increased plant resistance to pathogens.

RL-stabilized nanoemulsions appeared to have a significantly higher bioprotective effect than RLs suspensions. The main difference between the suspension and the nanoemulsion samples is the content of HD in the latter. This hydrophobic material may interact and disperse on the cuticular membrane on the plan cell surface. The cuticle layer consisting of cutin polyester and cuticular wax is the first barrier to protect the aerial parts of land plants from environmental stresses. The chemical composition of a plant cuticle can change in response to various abiotic or biotic stresses and plays essential functions in disease resistance responses. *Arabidopsis thaliana* mutants altered in cutin content are resistant to *Botrytis cinerea* through other defense pathways, different from those canonical immune ones (Aragón et al., 2021). Therefore, an alteration of the cuticle composition by incorporation of the HD contained in nanoemulsions may act synergically on RLs action as defense inducers.

Novel bioprotectans should not cause harmful effects over non-target organisms as are the soil microbiota members. In this regard, we did not observe deleterious effects over the growth of diverse microorganisms (bacteria, fungi) naturally present in the substrate where we grow the plants. Additionally, RLs applied as nanoemulsions did not negatively affect the *in vitro* growth of the beneficial bacteria *P. fluorescens*. This characteristic is relevant as the agricultural producers that might use bioprotectants, would probably apply them concomitantly with biostimulants, which usually contain beneficial bacteria in their microbial consortia.

Here, we demonstrated that the nanoemulsions of synthetic RLs maintained, and sometimes increased the capability of these compounds to act as resistance inducers, providing higher plant protection against an hemibiotrophic bacterial pathogen without exerting negative effects on the leaf tissues. The preventive treatment with synthetic RL-Est appeared to be more effective at potentiating plant defenses against the compatible bacteria Pst-DC3000 than RL-Nat. Intriguingly, this RL-Est did not trigger cell death or callose deposition and significantly reduced ROS production. At the molecular level, we could confirm an induction of PR1 transcription by both formulations of RL-Nat (suspension and nanoemulsion) which agrees with previous reports (Sanchez et al., 2012). Even though the behavior of the ICS1 marker gene has not been previously analyzed, we found that it appeared to be a more sensitive marker than PR1 to the effect of the different RLs here tested, independently of their formulations. This is interesting as this gene encodes for an enzyme responsible for the synthesis of SA, which accumulates in response to the attack of biotrophic and hemibiotrophic pathogens. RL-Est has already been described as highly effective in wheat against the hemibiotrophic fungus *Zymoseptoria tritici* [14] and in tomato against another fungus, *Botrytis cinerea*, a necrotroph [23]. This might imply that RL-Est, at the doses here used, is probably acting as a priming inducer (RL- Induced Resistance stimulus) of the SA-pathway while collaterally activating the MYC-branch of the JA pathway, of which VSP2 is a marker gene (Vos et al., 2015). To verify if RL-Est nanoemulsions activate priming, time-course experiments of markers’ gene expression after RL-Est application in mock and pathogen-infected plants should be performed. Additionally, the fitness costs of RL-Est use need to be evaluated. On the other hand, the other synthetic molecule studied here, RL-Suc, despite having a higher emulsifying power, showed low wettability. Accordingly, although it induced marker genes of the SA and JA pathway, it provided a less strong phytoprotection against Pst-DC3000 and was previously reported as unable to significantly inhibit *Zymoseptoria tritici* hemi-biotrophic fungal growth *in vitro* and *in planta* [5]. These characteristics make it less ideal for its use as a bioprotective nanocarrier.

In conclusion, synthetic mono-RLs containing a C12 acyl chain may have similar or enhanced surface activity and emulsifying power than RL-Nat, strongly depending on the chemical linker between the acyl chain and the rhamnose moiety. RL-Suc stood out as the best emulsifying amphiphile, surpassing the RL-Nat products. However, due to the drawbacks before mentioned, its poorly suited as a plant protective bio-carrier. On the contrary, the nanoemulsions of RL-Est provided better wettability and stability to the colloidal system, and were more effective RL-Nat at halting bacterial growth *in planta*, with almost no deleterious or collateral effects over the plant (no cell death, less ROS, no change in callose deposition and moderate levels of defense gene markers activation) making this preparation promising for future investigations to develop bio-carriers for phyto-protective hydrophobic compounds.

## Data availability statement

The raw data supporting the conclusions of this article will be made available by the authors, without undue reservation.

## Author contributions

Conceptualization: MLF, MM, GF. MCB performed the RLs surface activity assays. MM, JAVP and NEN developed the nanoemulsions. RVV performed the spectroscopic analysis. LC and PM synthesized and characterized the RL-Est and RL-Suc. MM performed the molecular structural analysis. NEN monitored the microbiological assays. GF, LTK, JAVP and MFB performed the tests in planta and against beneficial microbes of the RLs suspensions and nanoemulsions. MLF developed the wettability assays. MLF and GF wrote the manuscript. MM, MLF, LTK, MCB and MFB created figures and tables. All authors contributed to the article and approved the submitted version.
