# Supplementary material for: Nanoemulsions of synthetic rhamnolipids act as plant resistance inducers without damaging plant tissues or affecting soil microbiota
Source: Front Plant Sci. 2023 Aug 22;14:1195718. doi: 10.3389/fpls.2023.1195718 (PMC10478713; doi:10.3389/fpls.2023.1195718)
Supplement: Supplementary file 1 [file DataSheet_1.pdf]

Supplementary Results, Figures and Tables

Nanoemulsions of synthetic rhamnolipids act as plant resistance inducers without damaging plant tissues or affecting soil microbiota

Milagro Mottola, Maria Candelaria Bertolino, Lucille Tihomirova Kourdova, Jessica Aye Valdivia Pérez, María Florencia Bogino, Natalia E. Nocelli, Ludovic Chaveriat, Patrick Martin, Raquel Viviana Vico, Georgina Fabro and Maria LauraFanani

Supplementary Results:

1_^{1}H and ^{13}C NMR assignments of synthetic RLs

The chemical shifts are reported in parts per million (ppm), the residual solvent signal was taken as the reference [1].

Dodecanoyl α/β-L-Rhamnopyranoside (RL-Est)

^{1}H NMR (DMSO-d6, residual solvent signal 2.54 ppm): 5.80-4.82 (m, 2H), 3.81-3.64 (m, 4H), 3.30-3.25 (m, 2H), 3.20-3.13 (m, 2H), 2.38-2.31 (m, 4H), 1.55 (s, 4H), 1.27-1.16 (m, 38H), 0.89 (m, 6H, CH_3). ^{13}C NMR (DMSO-d6, residual solvent signal 40.45 ppm): 173.6, 172.6, 95.0, 92.1, 75.0, 74.6, 73.6, 71.3, 70.2, 69.2, 68.9, 68.7, 66.2.4, 34,7, 32.3-23.1 (CH_2 alkyl chain), 18.9, 18.6, 14.9.

Dodecyl α/β-L-Rhamnopyranoside (RL-Eth)

^{1}H NMR (DMSO-d6, residual solvent signal 2.54 ppm): 3.79 (m, 2H), 3.69 (m, 2H), 3.50-3.40 (m, 2H), 3.18-3.05 (m, 4H), 2.99–2.93 (m, 4H), 1.58-1.51 (m, 4H), 1.35-1.28 (m, 42H), 0.88 (m, 6H, CH_3). ^{13}C NMR (DMSO-d6, residual solvent signal 40.45 ppm): 103.7, 77.7, 74.4, 71.0, 69.5, 62.0, 32.2-29.6 (CH_2 alkyl chain), 24.4, 23.0, 14.9.

α/β-L- Rhamnopyranosyl N-dodecylcarbamate (RL-Car)

^{1}H NMR (DMSO-d6, residual solvent signal 2.54 ppm): 5.18-4.87 (m, 2H), 3.62-3.48 (m, 6H), 3.38 (m, 3H), 2.98 (m, 3H), 1.42 (m, 4H), 1.28 (m, 34H), 1.16 (m, 4H) 0.89 (m, 6H, CH_3). ^{13}C NMR (DMSO-d6, residual solvent signal 40.45 ppm): 154.9, 94.9, 72.0, 70,9, 70.7, 68.5, 41.8, 40.9, 33.2–23.0 (CH_2 alkyl chain), 21.7, 18.8, 14.9.

Dodecenylsuccinate α/β-L-Rhamnopyranoside (RL-Suc)

^{1}H NMR (DMSO-d6, residual solvent signal 2.54 ppm): 5.47 (m, 2H), 5.37(m, 2H), 4.91-4.82 (m, 2H), 4.08-4.04 (m, 2H), 3.81-3.56 (m, 4H), 3.53-3.29 (m, 4H), 2.90-2.85 (m, 2H), 2.58-2.53 (m, 4H), 2.02-1.94 (m, 8H), 1.32-1.17 (m, 34H), 0.89 (m, 6H, CH_3).^{13}C NMR (DMSO-d6, residual solvent signal 40.45 ppm): 176.3,174.1, 172.3, 134.0, 127.1, 94.9, 91.8, 75.1, 73,6, 72.3, 68.5, 60.7, 41.3, 36.3–22.9 (CH_2 alkyl chain), 18.8,18.4, 14.9.

[1] H.E. Gottlieb, V. Kotlyar, A. Nudelman, NMR Chemical Shifts of Common Laboratory Solvents as Trace Impurities, J. Org. Chem. 62 (1997) 7512–7515.

Supplementary Figure S1

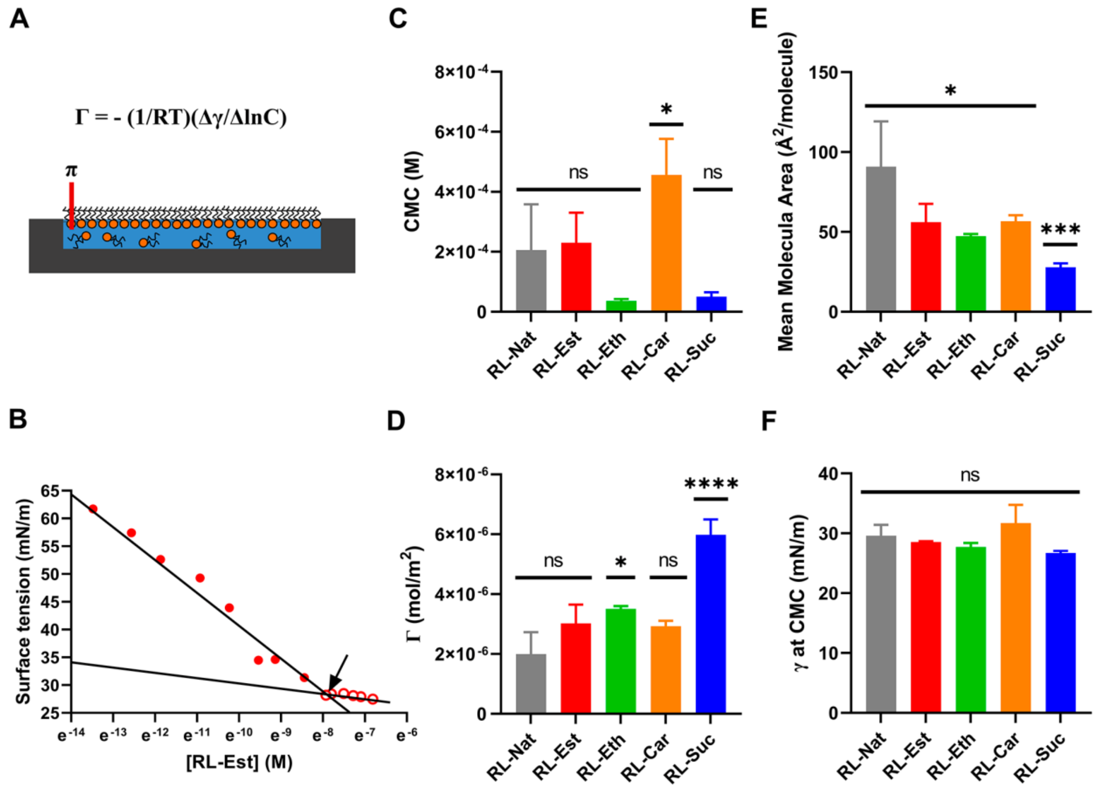

Supplementary Figure S1. Surface analysis of synthetic rhamnolipids. Representative examples of the surface activity studies performed and comparative plots of the outcome results. (A) Schematic representation of the Langmuir trough system for surface analysis and the Gibbs adsorption equation. The surface excess concentration (Γ) is a function of the slope in the surface tension decrease vs subphase surfactant concentration plot. (B) Representative surface tension study as a function of subphase surfactant concentration. The system follows the Gibbs adsorption equation at low surfactant concentrations, and above CMC (arrow), the surface tension becomes independent of surfactant concentration. (C-F) Comparative plot of surface analysis results for natural and synthetic RLs. The values are the mean of three independent experiments ± SD.

Supplementary Figure S2

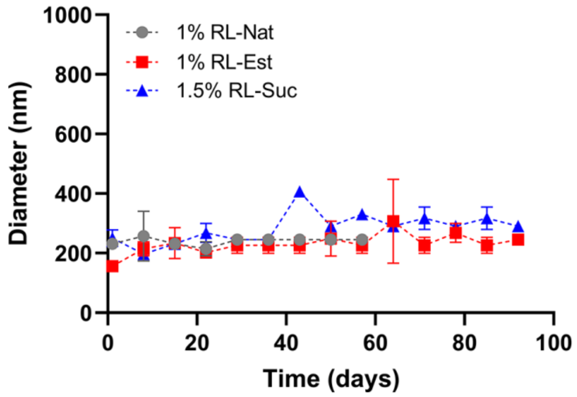

Supplementary Figure S2. Stability of nanoemulsions. DLS measurements were obtained for three months to verify the stability of the studied systems (see Table 2). The sample RL-Nat with 1% m/v of HD was stable for two months. The values are the mean ± SD from two independent samples.

Supplementary Figure S3

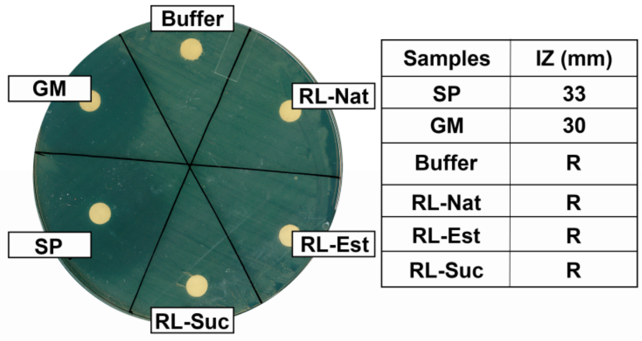

Samples	IZ (mm)
SP	33
GM	30
Buffer	R
RL-Nat	R
RL-Est	R
RL-Suc	R

Suplementary Figure S3. Effect of RLs nanoemulsions on the growth of *Pseudomonas syringae* pv tomato DC3000. The halo of the bacterial growth inhibition zone (left panel) and the inhibition zone (IZ) in mm (right panel) are shown. Known antibiotics were used as positive controls: SP (spectinomycin), GM (gentamycin). R: resistant.

Supplementary Table 1. List of primers used in this study.

Primer name	Target Gene	Sequence (5’ to 3’)
UBQ5-FW	AT3G62250	GTGGTGCTAAGAAGAGGAAGA
UBQ5-RV	AT3G62250	TCAAGCTTCAACTCCTTCTTT
PR1-FW	AT2G14610	ATGAATTTTACTGGCTATTCTC
PR1-RV	AT2G14610	AGGGAAGAACAAGAGCAACTA
ICS1-FW	AT1G74710	CTGCTGTAGAGAAGGCTTTAGAGATGA
ICS1-RV	AT1G74710	AGTCTCTCAGGCGTGTTTCCGAT
PDF1.2 FW	AT5G44420	TTCTGCTTTCGACGCA
PDF1.2 RV	AT5G44420	TGTCCCACTTGGCTTCTCG
VSP2 FW	AT5G24770	GAAGCCTAATGGTTCGAACTTG
VSP2 RV	AT5G24770	CACGAGACTCTTCCTCACCTTT
